# Are COVID-19 Vaccine Boosters Needed? The Science behind Boosters

**DOI:** 10.1128/jvi.01973-21

**Published:** 2022-02-09

**Authors:** Rachel M. Burckhardt, John J. Dennehy, Leo L. M. Poon, Linda J. Saif, Lynn W. Enquist

**Affiliations:** a American Academy of Microbiology, Washington, DC, USA; b Biology Department, Queens College and The Graduate Center of The City University of New York, New York, New York, USA; c School of Public Health & HKU-Pasteur Research Pole, LKS Faculty of Medicine, The University of Hong Kong, Hong Kong, China; d Center for Food Animal Health, Department of Animal Sciences, College of Food, Agricultural and Environmental Sciences, OARDC, The Ohio State University, Wooster, Ohio, USA; e Department of Molecular Biology, Princeton University, Princeton, New Jersey, USA; f Veterinary Preventive Medicine Department, College of Veterinary Medicine, The Ohio State University, Wooster, Ohio, USA; University of California, Irvine

**Keywords:** COVID-19, SARS-CoV-2, immunity, vaccines

## Abstract

Waning vaccine-induced immunity coupled with the emergence of SARS-CoV-2 variants has led to increases in breakthrough infections, prompting consideration for vaccine booster doses. Boosters have been reported to be safe and increase SARS-CoV-2-specific neutralizing antibody levels, but how these doses impact the trajectory of the global pandemic and herd immunity is unknown. Information on immunology, epidemiology, and equitable vaccine distribution should be considered when deciding the timing and eligibility for COVID-19 vaccine boosters.

The global impact of the COVID-19 pandemic has been unprecedented, but so too have the speed of development and efficacy of vaccines for COVID-19. Less than a year from first identifying SARS-CoV-2 as the causative agent of COVID-19, multiple safe and highly effective vaccines were authorized for use around the world. But the longevity of vaccine-induced immunity is unknown, sparking debate on the need for “booster” vaccine doses in the hopes these will increase protection from SARS-CoV-2 infection and disease. Fears of waning immunity and the emergence of variants of concern (VOC) have fueled this debate. But who should receive boosters and when are not easy questions to answer, especially given global vaccine distribution inequity. To make a decision, scientists must take into consideration known data on vaccines and immune memory, public health, epidemiology, and ethics for diverse age ranges and socioeconomic groups.

## TEXT

## SARS-CoV-2 VACCINES INDUCE ROBUST AND PROTECTIVE IMMUNE RESPONSES, EVEN AGAINST VIRAL VARIANTS

A plethora of vaccines against SARS-CoV-2 were developed in an unprecedented rapid time frame. They included conventional inactivated (Sinovac, Sinopharm), adenovirus (Ad)-vectored (Gamaleya, J&J Janssen, AstraZeneca), and mRNA (Pfizer, Moderna) vaccines. In the randomized clinical trials, their efficacy against severe disease ranged from 59 to >95% but was lower against mild or asymptomatic disease (∼47% to 94%) (1, 2). The mRNA vaccines showed consistently higher efficacy rates, but they elicited lower neutralizing antibody (NAb) titers in those >70 years of age at 6 months postvaccination (30% with no NAbs) (2, 3). In a Pfizer mRNA vaccine 6-month cohort in Israel, lower IgG Ab levels, especially in those >60 years, were correlated with subsequent infection (4).

Both the Ad vector and mRNA vaccines were engineered to express the prefusion spike protein (S) to induce NAbs. Both vaccine types stimulated effective innate immune responses (adjuvant effects) leading to enhanced adaptive immune responses. mRNA and Ad vector vaccines induced serum IgG S and receptor-binding domain (RBD) binding Ab and NAbs. These Ab responses generally were highly correlated, and the Abs persisted for at least 6 months (2, 3, 5). Primarily S-specific Th1 and T follicular helper (Tfh) CD4^+^ T cells, with lower CD8^+^ T cell responses and persisting memory T and B cells, were also documented in mRNA vaccinees (6). Emerging data from nonhuman primates (NHP) and vaccinees suggest that as for most other licensed vaccines, NAbs may be a potential correlate of protection (7–9).

Serum Abs are produced by short-lived plasmablasts, leading to the early peak of Ab responses that are then maintained by long-lived plasma cells (10–12). Multiple studies have documented 5- to 10-fold-decreased serum IgG Abs within 5 to 6 months after mRNA vaccination (10, 12), but serum IgG and NAbs were detectable for 10 months in mild COVID-19 cases (13). Data from long-term follow-up studies of vaccinated individuals are yet to be released, but B and T cell responses can still be detected in vaccinees for at least 6 months after the second dose (5, 14). Additionally, a recent study demonstrated that the durability of vaccine-induced B or T cell responses in COVID-19-recovered individuals was similar to that of uninfected vaccinated individuals (14). Moreover, after mRNA vaccination or natural infection, the memory B cells induced continued to evolve in germinal centers with increasing levels of somatic hypermutation and Abs of increased affinity. These memory B cells persist longer term and are essential for recall secondary Ab responses to protect against reinfection (5, 10–12, 14–16). Notably, memory B cell-expressed Abs had greater potency and breadth after natural infection compared with mRNA vaccination (10, 15, 16). This may explain in part why natural infection conferred a higher level of protection against S variants in some studies (10, 17). In contrast, an epidemiologic analysis of U.S. patients hospitalized with COVID-19-like illness and whose previous infection or mRNA vaccination occurred 90 to 179 days earlier found that convalescent but unvaccinated patients were 5.49-fold more likely to have laboratory-confirmed COVID-19 than mRNA vaccine recipients with no prior documented infection (19.75-fold higher if >65 years) (18). However, differences from the Israeli retrospective cohort study included protection against infection (any SARS-CoV-2-positive test) versus protection against hospitalization and a longer interval of 6 months versus 90 to 179 days since mRNA vaccination or infection, respectively.

Emergence of SARS-CoV-2 variants of concern (VOCs) with multiple substitutions in key Ab epitopes of the S glycoprotein may compromise immunity by partial escape from NAbs. Multiple studies suggest equivalent NAbs against the Alpha (B.1.1.7) variant but severalfold-reduced NAbs to the Gamma (P.1) and Delta (B.1.617.2) variants versus original strains in sera of vaccinated or recovered individuals. In most studies, NAb titers were lowest against the Beta (B.1.351) variant, but all variants were neutralized to a variable extent attributable to the polyclonal Ab responses induced (3, 12, 19). In contrast, T cell responses induced by mRNA vaccines or natural infection were not substantially affected by the variant substitutions (20). CD4^+^ T cells from recovered individuals mounted a broader antigen-specific response across the structural and accessory gene products, whereas the CD8^+^ T cells are predominantly nucleocapsid specific while spike-specific responses are substantially lower in frequency (20). Potentially these CD8^+^ T cells against the nucleocapsid (N) may also contribute to immunity after natural infection, since these N-specific responses, which are not induced by mRNA or Ad vector vaccines, may be more highly conserved against VOCs (20).

The half-life of SARS-CoV-2 NAbs in COVID-19-recovered individuals is a few months, depending on disease severity (12, 13, 19). Although these antibody responses wane rapidly, SARS-CoV-2-specific B and T cell memory responses can still be detected at least 1 year after the infection (12, 16, 21). Similar waning of antibody response can also be found in noninfected vaccinees. Of interest, modeling of genetic traits between SARS-CoV-2 and the 5 other human coronaviruses to estimate Ab decline and the probability of reinfection revealed short-lived immunity of 3 months to 5 years (median 16 months) after peak Ab titer (22). Waning of Ab-mediated protection and the emergence of variants are consistent with occurrence of reinfections after 8 months in convalescent individuals infected with the Gamma variant (23) and vaccine breakthrough infections by variants (4, 24). However, despite lower NAb titers against variants, mRNA and other vaccines protected against most hospitalizations and deaths, including those resulting from infection with the Delta variant (5, 25). Whether the variants contribute to reduced symptomatic protection in vaccinees (“breakthrough infections”) and convalescent individuals is an important unanswered question.

Those individuals with lower initial SARS-CoV-2 Ab responses (elderly, immunocompromised) and whose Ab responses have waned after 6 months may be at increased infection risk. The elderly, who are the most susceptible to COVID-19 disease and death, have more restricted Ab and naive T cell repertoires that likely constrain their immune responses (12). The increased transmissibility of the Delta variant along with the lower initial magnitude and waning Ab responses appears to have contributed to vaccine breakthrough infections, especially for severe cases in elderly people and those with comorbidities (4, 24) or immunocompromised individuals (26). These data prompted recommendations for booster vaccine doses for these vulnerable groups.

Importantly, infected vaccinees still shed and transmitted the virus, more so for the Delta than the Alpha variant (27), although for shorter times and with smaller amounts of infectious virus (28–30). Thus, virus transmission was reduced but not eliminated by the current vaccines. An explanation is that the current parenteral vaccines induce mainly serum IgG Abs, which are highly effective in prevention of systemic or lower respiratory tract (LRT) infections (reducing severe disease and hospitalizations) but less effective at blocking virus infection of the oral cavity and upper respiratory tract (URT) to prevent virus shedding and transmission. Studies of mRNA 1273 vaccine in challenged NHP confirmed that lower serum S Ab levels were required to reduce virus replication in the LRT than in the URT (7). Likewise, mRNA vaccinees had fewer S-specific T cells expressing longevity (CD127) or nasopharynx homing markers than did convalescent individuals (31). These findings, coupled with the time needed for antigen restimulation of vaccine-induced memory T and B cells to evoke secondary immune responses (12), may enable the highly transmissible variants to infect the URT and transit to the LRT in vulnerable patients or those with waning serum Abs, increasing disease risk. Future vaccines that induce broadly reactive mucosal IgA and serum IgG NAbs and augment T cell repertoires may provide enhanced protection against both disease and transmission after exposure to variants (32).

## KNOWN IMPACT OF SARS-CoV-2 VACCINE BOOSTER DOSES

Several publications have described the impact of SARS-CoV-2 vaccine booster on human immune responses (33–37). However, many studies primarily targeted specific groups (e.g., elderly or immunocompromised individuals) and analyzed different vaccine types. Some of these studies included only a small number of participants. With the exception of antibody neutralization, our understanding of immune correlates of SARS-CoV-2 protection is limited (7–9, 38). In addition, the minimal NAb level required for preventing infection or severe clinical outcomes and the importance of vaccine-induced memory B and T cells against SARS-CoV-2 are yet to be fully determined. Thus, the findings of these studies should be interpreted with caution and not extrapolated to the entire human population.

Different types of COVID-19 vaccines including mRNA, viral vector, inactivated, and protein-based vaccines are used as boosters (33, 36, 38–41). In general, a booster dose can substantially increase NAb levels or their surrogate markers (e.g., anti-spike IgG) against SARS-CoV-2. A booster dose also helps stimulate SARS-CoV-2 Abs in those who did not respond to a standard vaccine, such as immunocompromised individuals (29, 35, 42). The level of Abs with cross-reactivity against different SARS-CoV-2 VOCs is also enhanced by a booster (33, 36). This finding suggests that a high level of SARS-CoV-2-specific Abs maintained by repeated immunizations is sufficient to control COVID-19 caused by existing antigenic variants. Recent real-world data from Israel show that a third dose of an mRNA vaccine substantially reduced the risk of infection or severe illness caused by the Delta variant (33). Similarly, a recent large-scale study from Chile also showed that 2 doses of an inactivated vaccine followed by a third dose of an inactivated, mRNA, or adenovirus-based vaccine enhanced the vaccine-induced protection against SARS-CoV-2 (Chile Ministry of Health, unpublished data). In a preliminary study of 458 mix-and-match mRNA and Ad vector vaccine booster recipients, substantial increased NAb responses occurred in all recipients after homologous or heterologous booster vaccination, including against Delta and Beta variants, even in the absence of preboost NAb titers (39). Likewise, NHP boosted 6 months after the primary mRNA 1273 vaccination with either homologous mRNA 1273 or heterologous Beta variant (mRNA 1273Beta) developed broader NAb titers to the Beta and Delta variants and protection against Beta variant challenge (43).

Although the protective role of vaccine-induced T cell responses against COVID-19 is not clear, these responses are induced by different types of SARS-CoV-2 vaccines (5, 6, 14, 44). Cross-reactive T cell responses against different VOCs can be stimulated by vaccination (14), indicating that conserved epitopes are shared by SARS-CoV-2 antigenic variants. Such T cell responses can be further boosted by a third dose (36). A study on transplant patients showed that T cell responses can be detected in the majority of patients after a booster (37). Assuming these vaccine-induced T cell responses have a protective role against COVID-19, a third dose of vaccine might increase the chance of inducing an additional level of protection in immunocompromised patients or poor vaccine responders. Together, these data demonstrate that there are some short-term benefits of using a booster dose to enhance vaccine-protective effects in adults, especially for vulnerable populations.

Several studies have demonstrated that SARS-CoV-2 vaccine boosters are safe for adults (36, 45, 46). In these studies, adverse reactions induced by a booster of mRNA or Ad-vectored vaccine usually are mild and are comparable to those caused by the second dose of an mRNA vaccine. Thus far, there is no evidence that a COVID-19 vaccine booster shot can induce any unexpected adverse reactions or additional risk of having severe adverse reactions. Although there is no short-term safety concern for a booster dose, the long-term immune consequences of repeated immunizations are not known. Studies have shown that repeated administration of influenza vaccine boosters within a short time (e.g., 2 doses/year) may result in blunting of vaccine-induced antibody responses (47). As SARS-CoV-2 continues to evolve in humans, we do not know whether repeated immunization with the same antigens would induce undesirable immune imprinting effects, thereby skewing the breadth of our immune response in our future encounters with other novel SARS-CoV-2 antigenic variants. Some preliminary studies on vaccine-induced immune responses in COVID-19-recovered individuals argue against this possibility (14, 15); however, further characterization of Ab and T and B cell immune memory responses and their longevity in individuals who have received multiple doses of COVID-19 vaccines is still needed.

## HERD IMMUNITY IS ACHIEVED VIA GLOBAL VACCINATION

Vaccines not only protect individuals from infectious disease but also protect those who are unable to be vaccinated and those for whom vaccinations are less effective. That is, vaccines protect the community as well as the individual (48). This protection is called “herd immunity.” As the proportion of immune individuals in a population rises, the proportion of susceptible individuals declines, and consequently, the probability of an infected individual encountering a susceptible individual also declines. Theoretically, there is a threshold beyond which the proportion of immune individuals is so large that infected individuals are likely to recover before spreading infections to susceptible individuals. Once the herd immunity threshold is exceeded, the rate at which new infections are initiated will be insufficient to sustain the epidemic and eventually the epidemic will subside.

It is important to note, however, that the herd immunity threshold is not a fixed parameter. The herd immunity threshold will vary, even in the midst of an epidemic, according to the characteristics of the virus and the population in which it is spreading. Relevant virus properties may include infectivity, transmissibility, and durability outside a host. Important population parameters may include contact rates between individuals, behavioral interventions such as masking, availability of treatments, and population clustering. All of these characteristics can be subsumed under a single parameter termed R_0_ or R naught, which is defined as the expected number of new infections generated by an infected individual in a population of susceptible individuals (49). If R_0_ is >1, then an epidemic will persist, and conversely, if R_0_ is <1, then an epidemic will wane. The herd immunity threshold relates to R_0_ in that it must be equal to or greater than 1 − 1/R_0_ in order to arrest an epidemic (49). For example, if a virus’s R_0_ equals 4, then the herd immunity threshold equals 0.75, meaning that 75% of the population must be rendered immune to infection in order to stop this virus’s continuing transmission.

So when will we achieve herd immunity to SARS-CoV-2? How will vaccine boosters affect this timeline? To answer these questions, it is helpful to consider how R_0_ is embedded in an approach to epidemiological theory called compartmental models. In their simplest form, compartmental models assign individuals in a population to one of three categories: susceptible, infectious, and removed. Infectious individuals are generated when susceptible individuals are infected by a pathogen, which occurs at a transmission rate, β. Individuals are removed from the infectious compartment at a rate γ when they recover from infection, succumb to infection, or are quarantined. Vaccinated individuals transition directly from the susceptible compartment to the removed compartment and are included in the removal rate, γ. In deceptively simple terms, R_0_ is ≈β/γ  (49).

It must be recognized that β and γ vary over time and place. *β* may increase with virus evolution, as is seen with the highly transmissible SARS-Cov-2 Delta variant (50), or due to host behavioral changes such as reduced mask usage and reduced social distancing. Removal rate γ may increase with improved treatments and expanded vaccine access. Furthermore, except in cases of death, residence in the removed compartment is not permanent. Vaccines are not 100% effective, and breakthrough infections occur (51). Reinfections of individuals who have recovered from COVID-19 are also possible (52). Immunity, whether vaccine induced or natural, wanes. SARS-CoV-2 vaccine booster shots will reduce reversions to virus susceptibility (33).

As of the time of writing, there have been 250 million confirmed COVID-19 cases globally (https://covid19.who.int/), but this number is almost certainly an underestimate. Roughly extrapolating from U.S. data suggesting that 60% of infections have gone unreported (53), actual global cases may number ∼615 million. In addition, an estimated 4.02 billion people have received at least one dose of a SARS-CoV-2 vaccine (https://www.nytimes.com/interactive/2021/world/covid-vaccinations-tracker.html). Thus, the removed category may contain as many as 4.5 billion individuals, or ∼57% of the world population. Despite the difficulties estimating R_0_, as alluded to above, it would appear that the herd immunity threshold is approaching, but some wonder whether herd immunity to SARS-CoV-2 will ever be achieved (54). The United Kingdom may be an important indicator. Although 68% of the population is fully vaccinated and at least 13.5% have recovered from infection, the United Kingdom reported an average of 41,326 cases per day in the last week of October 2021, a number that has remained relatively stable since July 2021 (https://covid19.who.int/).

The United Kingdom situation reflects an important axiom regarding herd immunity: we won’t get there unless we *all* get there. No country can hope to escape the pandemic by itself. Concerningly, vaccine distribution has been uneven. Much of the Global South remains unvaccinated. Even in countries where availability is high, vaccine hesitancy limits full uptake. Slow or incomplete vaccine uptake prolongs the epidemic and exacerbates other issues, namely, waning immunity and pandemic fatigue. Remaining vigilant and engaging in protective behaviors are draining, and lapses become increasingly likely. Collectively, these factors permit continuing virus evolution and the accession of mutations enhancing virus transmission and evasion of antibody-mediated neutralization. New variants may emerge that negate our progress unless we ensure protection for the entire global community.

In record time, a multitude of safe and effective COVID-19 vaccines have been developed. Yet, as with most vaccines, immunity wanes, and this coupled with the emergence of SARS-CoV-2 variants has led to increases in breakthrough infections. Booster doses have been shown to be safe and increase SARS-CoV-2-specific neutralizing antibody levels that are cross-reactive to current VOCs. This is especially important for protecting elderly and immunocompromised individuals who are at highest risk for severe COVID-19.

However, current vaccines provide highly effective and sustained protection against COVID-19-related hospitalizations and deaths across age groups, indicating that a booster dose may not be needed for all fully vaccinated individuals. Additionally, reaching herd immunity requires equitable vaccine distribution and access worldwide. Resources spent on administering booster doses may prolong unequal global vaccine distribution as well as increase vaccine hesitancy. Public health officials and policy makers should take these factors into consideration when deciding what groups should receive boosters and when.
